# Humidity-Driven Chloride Protonation and Surface Enrichment on Natural Salt Surfaces near Deliquescence

**DOI:** 10.1021/acs.est.6c06363

**Published:** 2026-08-07

**Authors:** Kinga Szaló-Pál, Nicolas Fauré, Jie Chen, Ruiqi Man, Wan Wei, Luca Artiglia, Markus Ammann, Thorsten Bartels-Rausch, Zhijun Wu, Erik S. Thomson, Xiangrui Kong

**Affiliations:** † Department of Chemistry and Molecular Biology, Atmospheric Science, 3570 University of Gothenburg, Gothenburg SE-41390, Sweden; ‡ Department of Environmental Systems Science, ETH Zürich, Zürich 8092, Switzerland; § State Key Joint Laboratory of Environmental Simulation and Pollution Control, College of Environmental Sciences and Engineering, 12465 Peking University, Beijing 100871, China; ∥ Center for Energy and Environmental Sciences, 28498 Paul Scherrer Institute, Villigen PSI CH-5232, Switzerland

**Keywords:** time-resolved APXPS, surface solvation, chloride activation, HCl, predeliquescence, atmospheric chemistry

## Abstract

Natural salts are ubiquitous in saline environments and atmospheric aerosols, where their surface chemistry influences multiphase reactions and chlorine activation. The release of chloride as reactive chlorine species affects tropospheric oxidation and ozone chemistry, yet the molecular-level processes governing chloride speciation near deliquescence remain poorly understood. Here, we investigate humidity-driven interfacial chemistry of a natural chloride-rich salt using ambient-pressure X-ray photoelectron spectroscopy (APXPS) and near-edge X-ray absorption fine structure (NEXAFS) spectroscopy. By systematically varying relative humidity (RH) near the deliquescence threshold of MgCl_2_·6H_2_O, we observe reversible changes in chloride speciation prior to the formation of a fully deliquesced bulk phase. Hydration induces pronounced surface enrichment and redistribution of chlorine-containing species, accompanied by the emergence of molecular HCl at the interface. This enrichment increases the surface availability of HCl-like species, suggesting a greater potential for gas-phase release under fluctuating atmospheric conditions. Depth-resolved measurements show that these transformations are confined to the outermost nanometers and are fully reversible upon dehydration. Complementary NEXAFS measurements reveal the structural transition from a crystalline salt to a hydrated and ultimately aqueous phase. These results demonstrate that RH-driven phase transitions in hygroscopic components such as MgCl_2_ create reactive, transient interfaces that govern chloride chemistry and may enhance chlorine activation under atmospherically relevant humidity fluctuations.

## Graphical abstract

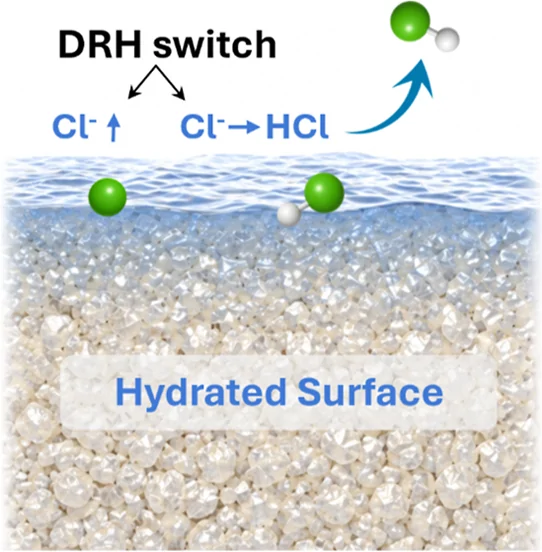

## 1 Introduction

Sea salt and mineral salts are ubiquitous components of atmospheric aerosols and natural saline surfaces.^1,2^ These materials play important roles in heterogeneous atmospheric chemistry by providing reactive interfaces where gas-particle interactions,^3^ ion redistribution,^4^ and multiphase reactions can occur. In particular, chloride-containing salts such as NaCl and MgCl_2_ are major constituents of sea spray aerosol and saline dust, and their surface chemistry strongly influences the release and cycling of reactive chlorine species in the atmosphere.^5^ Such processes affect tropospheric oxidation capacity, ozone chemistry, and secondary aerosol formation.

The physicochemical behavior of salts under varying relative humidity (RH) is often described in terms of hygroscopic phase transitions such as deliquescence and efflorescence. Deliquescence occurs when RH exceeds the deliquescence relative humidity (DRH), causing a crystalline salt to absorb water and form an aqueous solution. Conversely, efflorescence occurs when RH decreases below the efflorescence relative humidity (ERH), resulting in crystallization from a previously hydrated or aqueous phase. Because DRH and ERH can differ substantially, salts often exhibit hysteresis in their phase behavior during hydration and dehydration cycles. In natural systems, however, the response to RH changes may also be influenced by kinetic factors, including water-vapor transport, diffusion, and nucleation processes, in addition to the thermodynamic constraints defined by DRH and ERH.^6^ When RH exceeds the DRH, crystalline salts transform into aqueous brines, dramatically altering their chemical reactivity and transport properties. However, increasing evidence suggests that important transformations may already occur below the bulk DRH, where only limited amounts of water are adsorbed at the surface.^7–9^ Under these conditions, thin hydrated layers can form on salt surfaces, producing partially solvated interfacial environments that differ substantially from both dry crystalline solids and fully deliquesced solutions.^10^

Recent studies have demonstrated that such surface solvation can strongly modify the interfacial composition and reactivity of salts.^10–12^ For example, surface-sensitive spectroscopic measurements have revealed that certain ions redistribute upon hydration, leading to pronounced differences between surface composition and that of the underlying bulk solid or solution phase.^13–17^ In particular, magnesium chloride is known to exhibit strong hygroscopicity and to form hydrated surface layers at RH values well below its bulk DRH.^4,18^ These surface-hydrated states can influence ion mobility, interfacial electric fields, and the chemical speciation of halides.^19^ Related work on aqueous droplets and interfacial water has further shown that ion identity^20^ and pH can strongly modulate spontaneous interfacial redox chemistry, while the mechanistic role of interfacial electric fields remains an active topic of discussion.^21–27^

Despite these advances, the molecular-scale processes occurring during subdeliquescent hydration of natural salt mixtures remain incompletely understood. In particular, the fate of chloride ions during the transition from dry to partially hydrated surfaces remains largely unexplored, despite its importance for interfacial chemistry.^11,12^ Chloride protonation, which would lead to the formation of molecular HCl at the interface, could significantly influence chlorine activation pathways and gas-particle partitioning.^28^ However, direct spectroscopic evidence of such processes on natural salt surfaces is scarce. In particular, whether hydration induces interfacial ion enrichment that facilitates chloride protonation remains unclear.

Natural salt samples used in this study contain complex mixtures of ions commonly found in atmospheric mineral salts and playa aerosols.^29^ Such systems provide a more realistic representation of environmental particles compared to single-component laboratory salts, enabling investigation of interfacial processes under chemically complex conditions.^30,31^ In these mixtures, hygroscopic phase transitions are governed by the most deliquescent components, and in particular MgCl_2_ is expected to dominate the onset of hydration-driven transformations due to its low DRH.^4^ Accordingly, the deliquescence threshold discussed here is approximated by that of MgCl_2_·6H_2_O, which is expected to control the initial formation of a hydrated or liquid-like surface layer within the multicomponent system.

In this work, we investigate humidity-driven surface chemistry of a natural chloride-rich salt system using ambient-pressure X-ray photoelectron spectroscopy (APXPS) and complementary spectroscopic techniques. By systematically varying RH near the MgCl_2_-controlled hydration/deliquescence threshold, we probe changes in chloride speciation and surface composition associated with the formation of a thin hydrated interfacial layer. These observations highlight the importance of surface-confined hydration processes in controlling interfacial chemistry of natural salts and provide new insight into mechanisms governing chlorine activation at environmental interfaces.

## 2 Experimental Methods

### 2.1 Sample Collection and Preparation

Natural salt samples used in this study were collected from the Dalangtan (DLT) Playa in the Qaidam Basin, located on the northeastern Qinghai-Tibet Plateau, China.^29^ The Qaidam Basin is an arid region characterized by extensive evaporite deposits formed through long-term evaporation of saline lakes. The resulting salts contain complex mixtures of ions, including Cl^–^, SO_4_^2–^, Na^+^, and Mg^2+^, representative of mineral salts found in atmospheric dust and playa environments.^30^

Salt samples were collected directly from the playa surface and stored in sealed polyethylene containers. The samples were transported to the laboratory and stored at 4 °C prior to analysis. For chemical composition measurements, a portion of the salt was dissolved in ultrapure water (Milli-Q). The solution was filtered through a 0.22 μm membrane filter to remove insoluble particles before ion chromatography analysis. To prepare samples for spectroscopic measurements, aliquots of the salt solution were drop-cast onto conductive sample holders. The samples were subsequently heated to approximately 40 °C to accelerate solvent evaporation and formation of crystalline salt films. While ion pairing may reorganize during recrystallization, the overall ionic composition and dominant hygroscopic components are preserved, and the RH-dependent interfacial behavior is not expected to be significantly affected. The prepared samples were then transferred into the analysis chamber for measurements.

### 2.2 Chemical Composition Analysis

Bulk ionic composition of the salt samples was determined using ion chromatography (IC). Prior to analysis, the dissolved salt solutions were diluted to meet the detection range of the instrument. Anions (Cl^–^, SO_4_^2–^, Br^–^) and cations (Na^+^, K^+^, Mg^2+^, Ca^2+^, NH_4_^+^) were quantified using a Thermo Scientific IC system. Cation analysis was performed using a CS12A column (Dionex IonPac) with a methanesulfonic acid eluent, while anion analysis was performed using an AS11-HC column (Dionex IonPac) with potassium hydroxide as eluent.

### 2.3 Ambient-Pressure X-ray Photoelectron Spectroscopy (APXPS)

Surface chemical composition was investigated using APXPS at the ISS beamline of the Swiss Light Source (SLS) at the Paul Scherrer Institute (PSI), Switzerland. The SLS at the time of these measurements (2022) was a third-generation synchrotron radiation facility operating with an electron storage ring energy of approximately 2.4 GeV and providing high-brightness photon beams for spectroscopy experiments. The ISS beamline is a soft X-ray beamline optimized for surface and interface studies, delivering photon energies typically in the range of about 350–1500 eV for APXPS measurements.^32^

The APXPS system consists of an electron analyzer and a high-pressure prelens connected to a near-ambient-pressure cell that allows measurements of solid surfaces under controlled gas atmospheres at pressures up to several tens of millibar. Prior to experiments, the chamber was evacuated to a base pressure of approximately 10^–8^ mbar.

During measurements, the salt samples were exposed to controlled water vapor pressures. Water vapor was introduced into the near-ambient-pressure cell from a degassed water reservoir that underwent repeated freeze–pump–thaw cycles to remove dissolved gases. The RH at the sample surface was regulated by maintaining the water vapor pressure while controlling the sample temperature.

XPS spectra were recorded for several core levels including Cl 2p, O 1s, and other relevant elements. Both survey spectra and high-resolution spectra were acquired. Survey spectra were measured at a photon energy of 1000 eV to determine overall elemental composition. High-resolution spectra were acquired at selected photon energies to obtain detailed chemical-state information.

### 2.4 Depth-Resolved XPS Measurements

Depth-dependent information on surface composition was obtained by varying the kinetic energy of the emitted photoelectrons. The kinetic energy of the photoelectrons is related to the photon energy according to

1 KE=hν−BE−ϕ

where *h*ν is the photon energy, BE is the binding energy of the electron, and ϕ is the work function of the analyzer.

Higher kinetic energies correspond to larger inelastic mean free paths (IMFP) of photoelectrons, allowing electrons to travel longer distances before undergoing inelastic scattering. As a result, photoelectrons detected at higher kinetic energies originate, on average, from deeper regions of the sample. The effective probing depth, often described by the mean escape depth (MED), reflects contributions from both inelastic and elastic scattering processes.^33,34^

Depth-resolved information was obtained by varying the kinetic energy (KE) of the emitted photoelectrons through changes in photon energy. While higher KE increases the probing depth, XPS remains inherently surface-sensitive, probing only the outermost nanometers of the sample. The kinetic energies used in this study were approximately 300, 400, 600, and 700 eV, corresponding to increasing probing depths of the sample. The measurements were performed at an electron emission angle of 30° relative to the surface normal, which increases the surface sensitivity of the experiment. Based on the IMFP values calculated using the TPP-2 M model, the corresponding MED are approximately 0.9–1.0 nm (300 eV), 1.1–1.2 nm (400 eV), 1.5–1.6 nm (600 eV), and 1.7–1.8 nm (700 eV).^33^ Lower kinetic energies therefore probe the outermost surface region, while higher kinetic energies provide information integrated over slightly larger depths within the sample. By comparing elemental ratios obtained at these different probing depths, the distribution of species within the near-surface region can be evaluated.

### 2.5 Data Processing and Spectral Analysis

All XPS spectra were processed using background subtraction and peak-fitting procedures. Peak fitting was performed using Gaussian line shapes after subtraction of a linear background. Binding energies were calibrated using the C 1s peak of adventitious carbon at 284.8 eV.^35^ For elements exhibiting spin–orbit splitting (e.g., Cl 2p and S 2p), the separation and area ratios of the doublets were constrained according to known atomic parameters. Elemental intensities were normalized using photoionization cross sections and incident photon flux values. Elemental ratios were obtained by dividing the normalized peak areas of two elements measured at comparable probing depths. These ratios were used to evaluate changes in surface composition as a function of relative humidity and experimental conditions.

## 3 Results and Discussion

### 3.1 Surface-Bulk Composition and Interfacial Segregation

Understanding the relationship between bulk composition and surface chemistry is essential for interpreting interfacial processes in complex salt systems. Figure 1 shows the broadband XPS survey spectrum of the DLT salt mixture measured at a photon energy of 1000 eV. The spectrum exhibits characteristic photoemission peaks corresponding to O 1s, C 1s, Cl 2p, Cl 2s, S 2p, S 2s, Mg 2p, and Mg 2s, confirming the presence of chloride-, magnesium-, sulfur-, and oxygen-containing species at the outermost surface. The Cl 2p signal indicates the presence of chloride salts, while the O 1s peak likely arises from oxygen in sulfate species as well as residual or structurally bound oxygen-containing species (e.g., hydroxyl groups). The C 1s signal is comparable to the adventitious carbon commonly observed on air-exposed samples and does not indicate a substantial organic surface coating. Previous APXPS measurements on natural evaporite salts from the Qaidam Basin showed that the carbonaceous species are consistent with typical adventitious carbon and do not exhibit significant changes during hydration or with probing depth. Furthermore, the humidity-dependent Cl 2p transformation is reproduced in pure MgCl_2_ (Figure S1), suggesting that the observed effect does not require natural organic matter. While minor organic constituents may be present, the available evidence indicates that the RH-dependent chlorine chemistry is dominated by the inorganic MgCl_2_-rich surface layer rather than by surface-active organic species.

**Figure 1:**
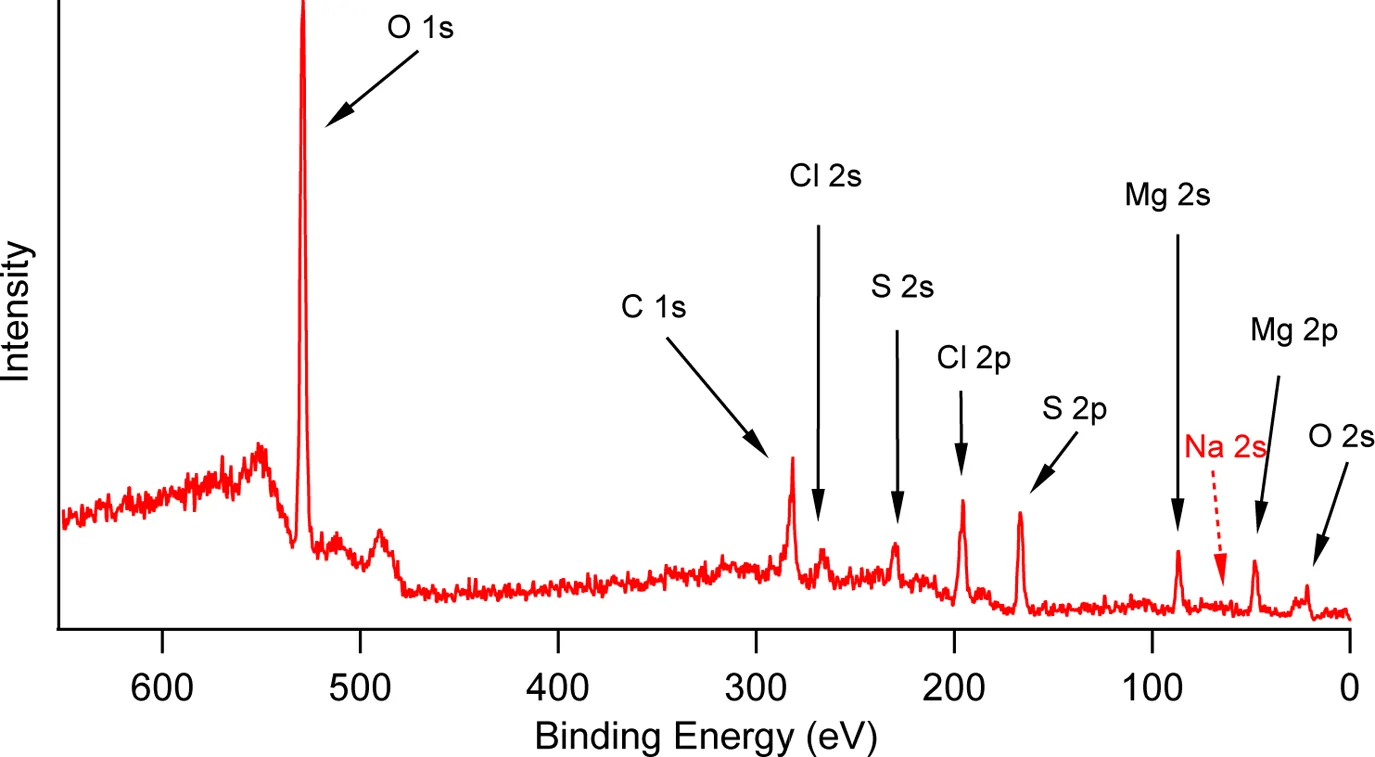
Broadband XPS survey spectrum of the DLT salt sample measured at a photon energy of 1000 eV. Measurements were performed at 0% RH with 0.47 mbar Ar introduced to mitigate charging effects during photoemission. Major photoemission peaks corresponding to O, C, Cl, S, and Mg are labeled. The expected position of the Na 2s signal is indicated (red), highlighting the absence of a detectable sodium contribution at the surface.

The bulk ionic composition of the salt, determined by ion chromatography (Table 1), is dominated by chloride, sodium, magnesium, and sulfate ions (pH = 6.4), reflecting the multicomponent nature of the natural system. The presence of Cl, Mg, and S signals in the XPS spectrum is consistent with these major ionic constituents.

**Table 1:** Ionic Composition of the Aqueous Extract of DLT Salt Measured by IC

	concentration (10^–3^ mol/L)							
pH	Cl^–^	Br^–^	SO_4_^2–^	Na^+^	NH_4_^+^	K^+^	Mg^2+^	Ca^2+^
6.4	4280.3	2.6	657.3	1600.7	26.9	342.7	1856.8	2.2

A notable discrepancy, however, is observed for sodium. Despite being a major component in the bulk, no clear sodium-related signal is detected in the XPS survey spectrum. The expected position of the Na 2s peak is indicated in Figure 1, but no distinct signal is resolved, likely due to its low photoionization cross section and sequential crystallization during drying, which can deplete Na at the surface.^4^ This suggests that sodium is depleted at the outermost surface, whereas magnesium-containing species are preferentially present within the few-nanometer probing depth of XPS.

Because APXPS probes only the outermost nanometers of the sample, surface composition is more relevant than bulk composition for interpreting the observed interfacial chemistry. The difference between the bulk IC composition and the XPS survey spectrum indicates a surface dominated by MgCl_2_-rich domains rather than bulk-representative material. High-resolution C 1s analysis (Figure S2) shows a spectrum dominated by the characteristic components of adventitious carbon (C–C/C–H, C–O, C=O and O–C=O), with no detectable carbonate component near 290 eV. Furthermore, the humidity-dependent Cl 2p transformation is reproduced in pure MgCl_2_ (Figure S1), indicating that the observed RH-dependent chlorine chemistry is governed primarily by the inorganic MgCl_2_-rich surface layer rather than by surface-active organic species.

Such surface-bulk differentiation is consistent with previous observations in mixed salt systems, where divalent cations such as Mg^2+^ exhibit stronger hydration and interfacial interactions, promoting their enrichment at or near the surface.^36,37^ The results therefore indicate that the surface composition of the natural salt mixture is not representative of the bulk, but instead is governed by interfacial ion redistribution processes that favor MgCl_2_-rich surface domains.

### 3.2 Humidity-Driven Transformation and Enrichment of Surface Chlorine Species

The surface of the salt mixture responds sensitively to changes in relative humidity, exhibiting pronounced transformations in chlorine speciation as RH approaches the deliquescence threshold. Time-resolved APXPS measurements reveal that these changes occur within a narrow RH range near the onset of hydration. Figure 2a presents the time-resolved Cl 2p spectra together with the RH trajectory, where the color scale represents the spectral intensity. The dashed line marks the DRH of MgCl_2_·6H_2_O (33% RH), the thermodynamically stable hydrate of MgCl_2_,^18,38^ used here as a reference for the onset of hydration. In mixed salt systems, effective deliquescence may occur at lower RH due to interactions between components, leading to partial or stepwise solution formation. This reference is included because MgCl_2_ has a substantially lower DRH than other major salts present in the system and because previous surface-sensitive measurements, together with the XPS results presented here, indicate that the outermost surface is dominated by MgCl_2_-rich domains despite the multicomponent bulk composition. The RH-dependent transformation observed here should therefore be interpreted as hydration of a MgCl_2_-dominated interfacial layer rather than deliquescence of a pure MgCl_2_ phase. Figure 2b–g show representative Cl 2p spectra corresponding to the different RH stages. The shaded regions in Figure 2a denote periods after RH changes during which the Cl 2p spectra continued to evolve and were therefore excluded from steady-state interpretation. Apparent constant RH does not necessarily imply sample equilibration, as water uptake may be limited by vapor transport, diffusion, and dissolution/crystallization kinetics. Representative spectra used in the analysis were selected only after stabilization of both the environmental conditions and the Cl 2p spectral response. The trajectories of pressure and temperature during the experiment are provided in the Supporting Information.

**Figure 2:**
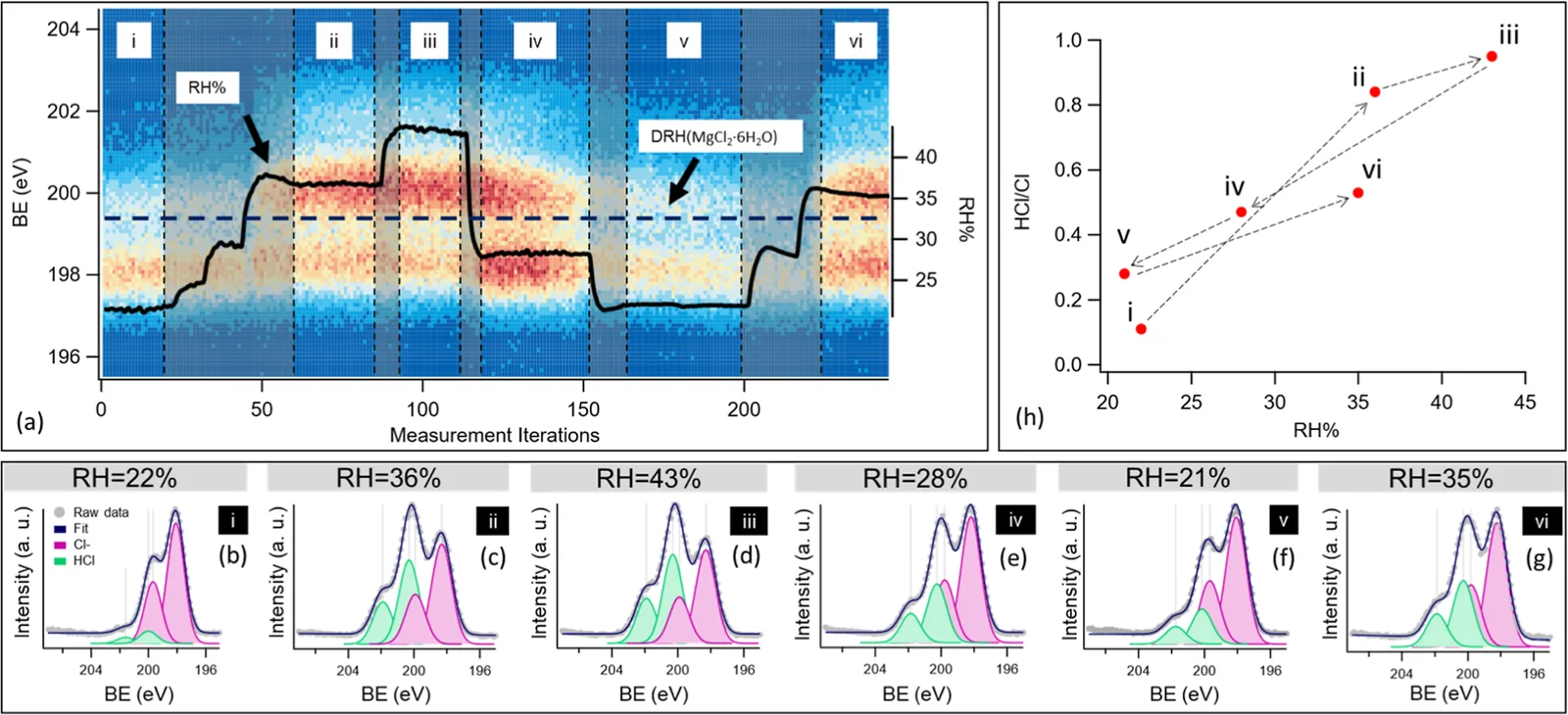
(a) Time-resolved Cl 2p XPS spectra displayed as a function of measurement iteration, together with the corresponding RH trajectory (black line). The color scale represents spectral intensity. The dashed line indicates the DRH of MgCl_2_·6H_2_O (33% RH). Shaded regions denote transient periods following RH changes before stabilization. (b–g) Representative Cl 2p spectra corresponding to selected RH stages indicated in panel (a). (h) HCl/Cl^–^ intensity ratio obtained from spectral fitting, plotted as a function of RH. Uncertainty in the HCl/Cl^–^ ratio, estimated from propagation of the peak-fitting uncertainties, is approximately 2% for a representative hydrated spectrum. Fitting parameters for the representative spectrum at RH 36% are provided in Table S1.

As shown in Figure 2a, the RH increases from ∼22% to ∼36%, accompanied by a clear change in the Cl 2p spectra and a corresponding increase in the HCl/Cl^–^ ratio (Figure 2h). Representative spectra recorded at these two RH conditions are shown in Figure 2b,c. Upon hydration, an additional spectral component appears at higher binding energy (green doublet in Figure 2c), indicating the formation of a second chlorine-containing species. The original Cl^–^ 2p_3/2_ peak is centered at ∼198.1 eV, while the new component appears at ∼200.2 eV, consistent with the formation of molecular HCl. This new spectral component is most likely assigned to molecular HCl formed at the hydrated salt surface.^39,40^ Although a direct comparison with a pure HCl reference spectrum was not available, the observed ∼2.0 eV chemical shift, reversible RH dependence, surface localization, and consistency with previous XPS studies of HCl at ice and aqueous interfaces support assignment of the new component to molecular HCl. We also explicitly discuss alternative interpretations, including chloride hydration and oxychlorine species, and acknowledge that the present APXPS data alone do not unambiguously distinguish among all possible assignments. Nevertheless, the combination of the observed chemical shift, its reversible humidity dependence, and its confinement to the hydrated surface layer makes molecular HCl the interpretation most consistent with the complete set of experimental observations. The APXPS environment may also influence HCl partitioning. Under the near-ambient-pressure conditions used here, interfacial HCl can remain in dynamic equilibrium with the hydrated salt surface because of the confined gas environment and continuous water-vapor exposure, whereas in the open atmosphere dilution and gas-phase transport would favor volatilization once HCl is formed. The present measurements therefore provide evidence for the formation and surface enrichment of an HCl-like interfacial species, but they do not directly quantify net gas-phase HCl release.

The proton required for HCl formation likely originates from interfacial water. Possible contributing mechanisms include Mg^2+^-associated hydrolysis within the concentrated hydrated layer and interfacial ion partitioning that favors proton and chloride accumulation relative to strongly hydrated Mg^2+^ ions. The present data do not allow these contributions to be distinguished. The two components are separated by ∼2.0 eV, consistent with previous observations for HCl at ice and aqueous interfaces, where similar ∼2 eV chemical shifts have been attributed to the neutral (covalent) Cl character of molecular HCl. Hydration of chloride (e.g., Cl^–^···H_2_O interactions) could increase the Cl 2p binding energy, but such effects are typically much smaller than the ∼2.0 eV separation observed here and are unlikely under the low RH conditions where only limited water is available. In addition, although oxidized chlorine species (e.g., ClO^–^) can exhibit comparable or larger chemical shifts relative to Cl^–^, their formation and interconversion would typically involve more complex and less readily reversible processes. The observed reversible evolution of the additional spectral component with RH, together with its emergence only upon hydration near the deliquescence threshold, is therefore inconsistent with oxychlorine formation and instead supports assignment to molecular HCl.^41^ Earlier work related to HCl on ice and calculated core-level binding energy shifts further support the assignment to HCl.^39,40,42^ The pronounced RH dependence further suggests that the observed behavior is linked to the formation of a concentrated hydrated interfacial layer near the MgCl_2_-controlled hydration transition, rather than representing a generic property of the air–water interface. Potential beam-induced effects were also considered. The ∼200.2 eV component appeared and disappeared reproducibly with RH cycling and largely returned to its initial state upon dehydration despite continuous X-ray exposure. This behavior is inconsistent with progressive beam damage and indicates that hydration is the primary driver of the observed spectral changes, although a synergistic contribution from X-ray irradiation and water vapor cannot be completely excluded. As RH increases further to ∼43% (Figure 2d), no significant changes are observed in the Cl 2p spectra, indicating that the interfacial chemical environment has already been established during the preceding hydration step.

The RH is subsequently decreased from ∼43% to ∼28%, as shown in Figure 2e. The intensity of the HCl doublet correspondingly decreases, consistent with the loss of interfacial hydration and reflected in a decrease of the HCl/Cl^–^ ratio (Figure 2h). This decrease likely reflects reversible proton transfer within the interfacial layer, where the proton is stabilized by the water network or by Mg^2+^-associated hydrolysis. While partial desorption of HCl to the gas phase cannot be excluded, the present data primarily indicate a dynamic equilibrium between interfacial HCl and chloride. In contrast, the Cl-doublet becomes more intense than in earlier spectra at similar RH conditions (∼40th measurement iteration). This behavior suggests a transient enrichment of chloride at the surface, potentially associated with changes in interfacial speciation during dehydration. A direct comparison with other elements (e.g., O, Mg, Na, C) is not available for this time-resolved data set, as tracking different core levels requires changes in photon energy and would interrupt the temporal evolution of the Cl 2p signal. The observed response may therefore reflect either partial deprotonation of interfacial HCl or a dynamic balance between HCl formation and loss (e.g., via desorption or beam-induced processes), rather than a purely reversible conversion. Such reversible transformations imply that simple RH cycling can redistribute chloride toward the particle surface, potentially increasing the availability of HCl-like species at the interface upon renewed hydration. In atmospheric environments, where aerosol particles frequently experience humidity fluctuations, this mechanism may influence chloride speciation during the aging of sea-salt and saline mineral aerosols.^5,43^

As RH decreases further to ∼22%, the Cl 2p spectra largely return to the initial state observed at the beginning of the experiment, confirming that the hydration-induced transformation is substantially reversible. When RH is subsequently increased again above the DRH of MgCl_2_·6H_2_O near the end of the experiment, the spectral response reappears, demonstrating the reproducibility of the RH-driven surface transformation.

The RH threshold for the observed transformation coincides with the DRH of MgCl_2_·6H_2_O, suggesting that this hydrate phase plays a key role in activating the surface chemistry. In contrast, other salts present in the system, such as MgSO_4_ or Na-containing salts, are unlikely to govern the transformation, as their DRH values are substantially higher than the RH range investigated here. This interpretation is further supported by measurements on pure MgCl_2_ (Figure S1), which exhibit analogous humidity-dependent changes in the Cl 2p spectra. In addition, Na-related signals are largely absent in the surface-sensitive XPS measurements, indicating a Na-depleted outermost layer. Previous studies have shown that MgCl_2_ can preferentially coat NaCl in mixed particles, supporting the presence of MgCl_2_-rich surface domains.^18^

Overall, these observations show that RH-driven hydration across the deliquescence threshold of MgCl_2_ induces reversible changes in chloride speciation at the salt surface. The formation and disappearance of HCl, together with pronounced surface enrichment and redistribution of chloride, indicate the formation of a chemically distinct interfacial layer associated with a thin, highly concentrated MgCl_2_ solution. This behavior is reproducible across multiple RH cycles (Figures S3–S5). Under atmospheric conditions, where particles frequently cross such phase transitions, this interfacial restructuring may increase the interfacial availability of chlorine-containing species relevant to chloride activation and HCl release. The hydration-driven enrichment of chloride at the interface, combined with interfacial HCl formation, implies that RH fluctuations may enhance HCl volatilization by increasing its concentration and accessibility at the surface.

### 3.3 Depth-Resolved Evidence for Interfacial Chlorine Enrichment and HCl Formation

To further examine the spatial distribution of the newly formed HCl species, depth-resolved XPS measurements were performed. Figure 3 shows the elemental ratios derived from these measurements, including (a) C*l*_tot_/Mg, (b) HCl/Cl^–^, (c) C*l*_tot_/O, and (d) Mg/O, measured at two RH conditions, 28% and 37%. The variation of these ratios with kinetic energy (KE) reflects the depth sensitivity of the measurement, where lower KE corresponds to higher surface sensitivity. A pronounced change in the elemental ratios occurs when RH increases from 28% to 37%, consistent with the formation of an additional Cl species under hydrated conditions.

**Figure 3:**
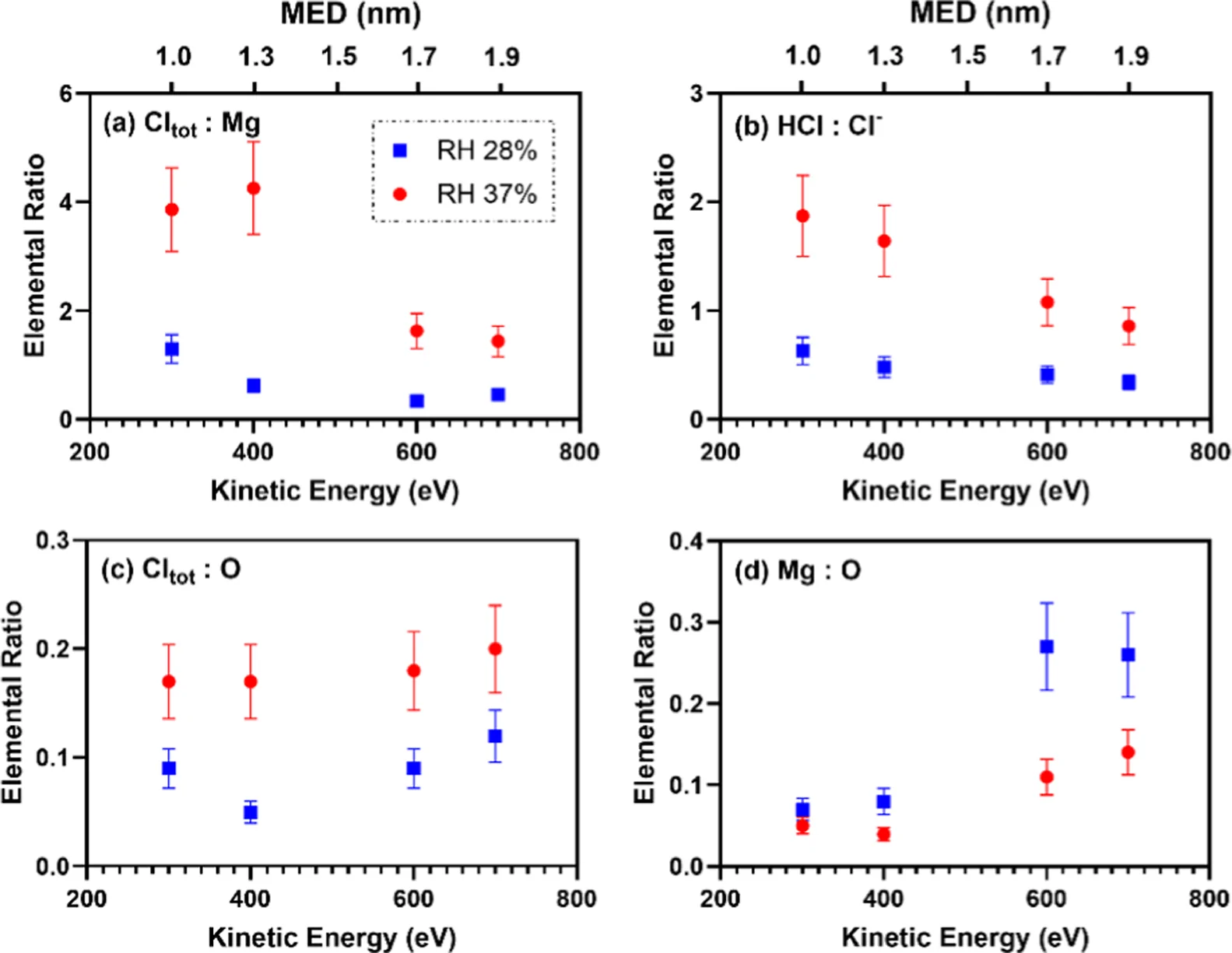
Depth profiles of elemental ratios derived from XPS measurements: (a) C*l*_tot_/Mg, (b) HCl/Cl^–^, (c) C*l*_tot_/O, and (d) Mg/O. In panels (a,c), C*l*_tot_ represents the total chlorine signal (Cl = Cl^–^ + HCl). Measurements were performed under two RH conditions, 28% and 37%. A clear change in the elemental ratios is observed when RH increases from 28% to 37%, consistent with the appearance of an additional chlorine-containing species at higher RH. Error bars represent uncertainties propagated from the XPS peak-fitting procedure.

As shown in Figure 3a, the C*l*_tot_/Mg ratio increases markedly at RH 37%, particularly at the two most surface-sensitive kinetic energies. This trend demonstrates strong surface enrichment of chlorine-containing species upon hydration, particularly within the outermost nanometers. Although restructuring of the hydrated surface layer and changes in photoelectron attenuation may influence the magnitude of the measured intensity ratios, these effects alone do not readily explain the consistent RH-dependent trends observed across multiple elemental ratios and probing depths. In particular, the stability of the Cl/O ratio under constant RH conditions indicates that spectra were acquired after equilibration, supporting the interpretation that the observed changes primarily reflect humidity-induced interfacial redistribution rather than continued water uptake during the measurements. Such enrichment indicates that hydration drives ion partitioning toward the interface, leading to preferential accumulation of chlorine-containing species in the outermost layers. Differential ion mobility may also contribute to the observed Cl/Mg separation: Cl^–^ and H^+^ are expected to redistribute more readily within the hydrated interfacial layer than strongly hydrated Mg^2+^. However, this mobility effect is likely coupled to surface solvation, Mg^2+^ hydration/complexation, crystallization history, and interfacial ion partitioning, so diffusion alone cannot be isolated as the sole driver of the observed enrichment.

Figure 3b further shows that the HCl/Cl^–^ ratio increases significantly at RH 37%, confirming the formation of molecular HCl upon surface hydration. The stronger enhancement at lower kinetic energies indicates that HCl is preferentially located at the outermost surface. This observation suggests that protonation of chloride occurs primarily at the hydrated interface.

A similar enrichment of chlorine relative to oxygen is observed in Figure 3c, where the C*l*_tot_/O ratio increases when RH rises from 28% to 37%. This behavior is notable because water uptake at higher RH should increase the oxygen signal due to condensed phase water. The observed increase in C*l*_tot_/O therefore indicates that the surface hydration process promotes a stronger enrichment of chlorine-containing species than the increase in oxygen associated with hydration.

Figure 3d shows that the Mg/O ratio decreases at RH 37%, indicating a relative enrichment of oxygen-containing species compared with magnesium after surface solvation. The relatively modest change suggests that the hydrated layer remains highly concentrated, consistent with a thin MgCl_2_-rich brine rather than a dilute aqueous phase. At RH 28%, the Mg/O ratio increases at higher kinetic energies (600–700 eV), suggesting that oxygen-containing species are concentrated mainly near the surface, while deeper layers contain relatively less oxygen.

These depth-resolved measurements indicate that RH-driven deliquescence of MgCl_2_ leads to a redistribution of ions within a thin, highly concentrated solution layer at the surface. Chloride species become enriched at the interface and are partially converted to molecular HCl, while oxygen-containing species increase due to water uptake. These results highlight the dynamic nature of the concentrated MgCl_2_-rich interfacial layer and show that crossing the hydration/deliquescence threshold induces significant changes in interfacial composition and speciation.

### 3.4 Structural Evolution during Hydration and Deliquescence Probed by NEXAFS

The NEXAFS spectra shown in Figure 4 provide complementary insight into the structural evolution of the salt during the transition from a dry crystalline state to a fully deliquesced aqueous phase. While the APXPS measurements primarily probe changes in surface composition and chloride speciation, NEXAFS is sensitive to the local coordination environment of the constituent elements and therefore provides information on structural changes associated with hydration.

**Figure 4:**
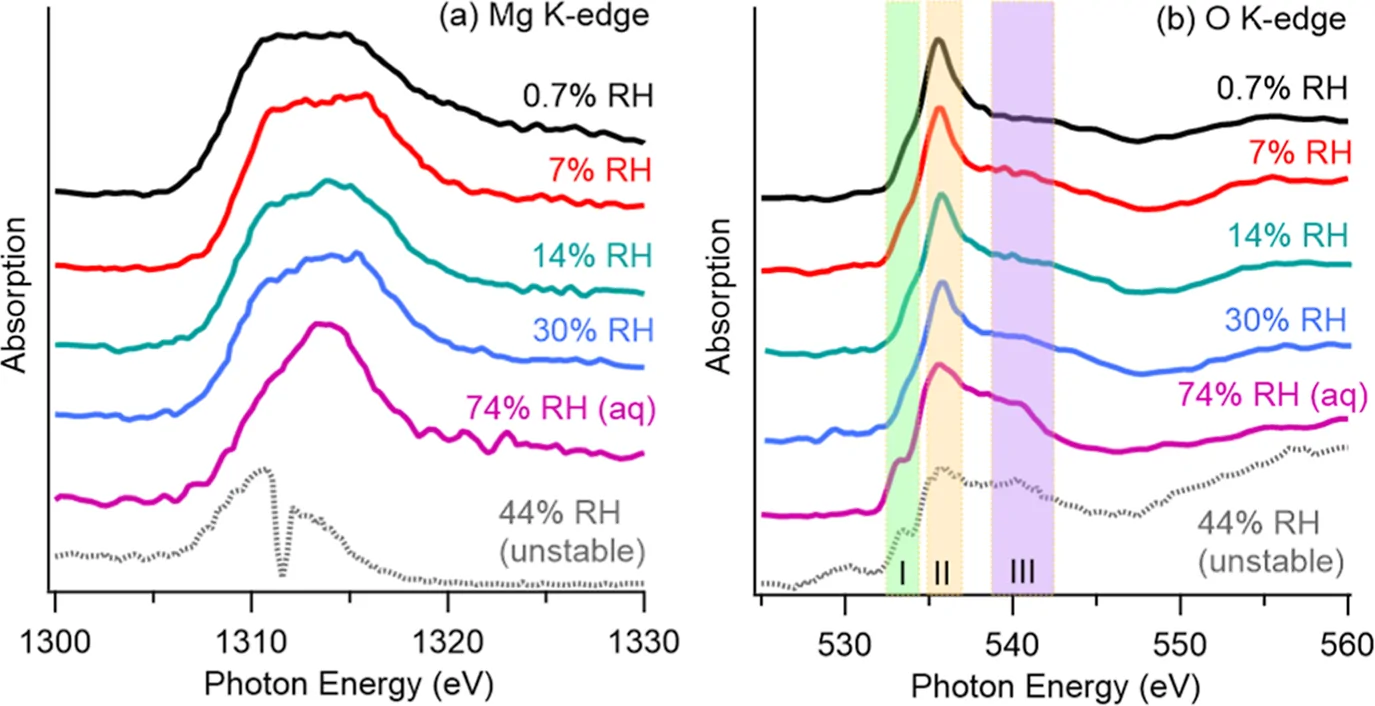
NEXAFS spectra recorded at (a) the Mg K-edge and (b) the O K-edge under different RH conditions, as indicated in the panels. At RH ≤ 30%, no deliquescence occurs and the salt remains in a solid phase. At RH 74%, full deliquescence has taken place and the sample is present as an aqueous solution. Measurements were also attempted at RH 44%, corresponding to the surface-solvation regime; however, the spectra obtained under these conditions were unstable and are therefore not considered reliable.

Figure 4a presents the Mg K-edge spectra measured at different RH conditions. At low RH (≤30%), the spectra exhibit well-defined features characteristic of crystalline MgCl_2_ hydrates, reflecting the ordered coordination environment of Mg^2+^ ions in the solid phase.^11^ As RH increases, the spectral features become progressively broader and less structured, indicating increasing hydration and structural disorder around Mg^2+^ ions. Similar spectral evolution has been reported for hydrated MgCl_2_ systems, where increased coordination by water leads to loss of long-range order and broadening of Mg K-edge features.^44^ At RH 74%, where full deliquescence occurs, the spectral profile becomes substantially smoother, consistent with Mg^2+^ ions being predominantly solvated in an aqueous environment. While the overall trends agree with previous APXAS studies of MgCl_2_-water interfaces, the spectral features differ in detail, likely reflecting differences in temperature, concentration, and interfacial versus bulk sensitivity.

The O K-edge spectra shown in Figure 4b further illustrate the progressive hydration of the salt. The spectra can be divided into three characteristic regions (I–III), corresponding to distinct oxygen electronic states. At low RH, the spectral features primarily originate from oxygen atoms associated with sulfate species and structural water within the crystalline salt.^37^ With increasing RH, the spectral shape gradually evolves, reflecting increasing contributions from hydrogen-bonded and adsorbed water molecules. At RH 74%, the spectral profile closely resembles that of liquid water, confirming the formation of a fully hydrated aqueous phase after deliquescence.

Measurements were also attempted at RH 44%, corresponding to conditions near the hydration transition identified in the APXPS experiments. However, the sample surface was visibly moving during the measurements, resulting in continuously changing spectral intensities and nonreproducible spectra. This behavior indicates that the surface was in a highly dynamic state near the onset of hydration and HCl formation. Although a representative NEXAFS spectrum could not be reliably obtained under these conditions, the observed instability itself is consistent with substantial restructuring of the partially deliquesced surface layer. As a result, these spectra were not considered suitable for quantitative interpretation.

Taken together, the NEXAFS and APXPS measurements demonstrate that significant interfacial transformations occur on natural chloride-rich salt surfaces as RH approaches the deliquescence threshold of MgCl_2_. RH-driven hydration induces reversible changes in chloride speciation, including the appearance of an additional chlorine-containing component most consistent with molecular HCl within a thin, highly concentrated hydrated surface layer. Depth-resolved measurements further reveal redistribution and enrichment of chlorine-containing species at the outermost interface. The observations indicate that the MgCl_2_ deliquescence threshold functions as a humidity-controlled switch, reversibly activating interfacial chloride redistribution and the appearance of HCl-like species. Under atmospheric conditions, where aerosol particles frequently experience RH fluctuations, such interfacial cycling may influence chloride speciation, chlorine activation, and gas-particle partitioning during aerosol aging.

## Supplementary Material



## Notes

High-resolution Cl 2p XPS spectra of MgCl2 under varying RH conditions; Cl 2p peak-fitting parameters and fit-quality metrics for a representative hydrated spectrum; time-resolved APXPS measurements during RH cycling; experimental time series of RH, temperature, chamber pressure, and Cl 2p spectral evolution; definition of transient and steady-state regions used in the analysis (PDF)
