# Supplementary material for: Humidity-Driven Chloride Protonation and Surface Enrichment on Natural Salt Surfaces near Deliquescence
Source: Environ Sci Technol. 2026 Aug 7;60(32):22791–800. doi: 10.1021/acs.est.6c06363 (PMC13492281; doi:10.1021/acs.est.6c06363)
Supplement: Supplementary file 1 [file es6c06363_si_001.pdf]

Supporting Information

# Humidity-Driven Chloride Protonation and Surface Enrichment on Natural Salt Surfaces near Deliquescence

Kinga Szaló-Pál[1], Nicolas Fauré[1], Jie Chen[2], Ruiqi Man[3], Wan Wei[1], Luca Artiglia[4], Markus Ammann[4], Thorsten Bartels-Rausch[4], Zhijun Wu[3], Erik S. Thomson[1], and Xiangrui Kong[1]

**Contents**

Pages: S1-S7
Figures: 4 (Figures S1-S4)
Tables: 1
Description:
Figure S1. High-resolution Cl 2p XPS spectra of $MgCl_2$ under varying RH.
Figures S2-S4. Time-resolved APXPS measurements during RH cycling, including RH, temperature, pressure, and Cl 2p spectral evolution.

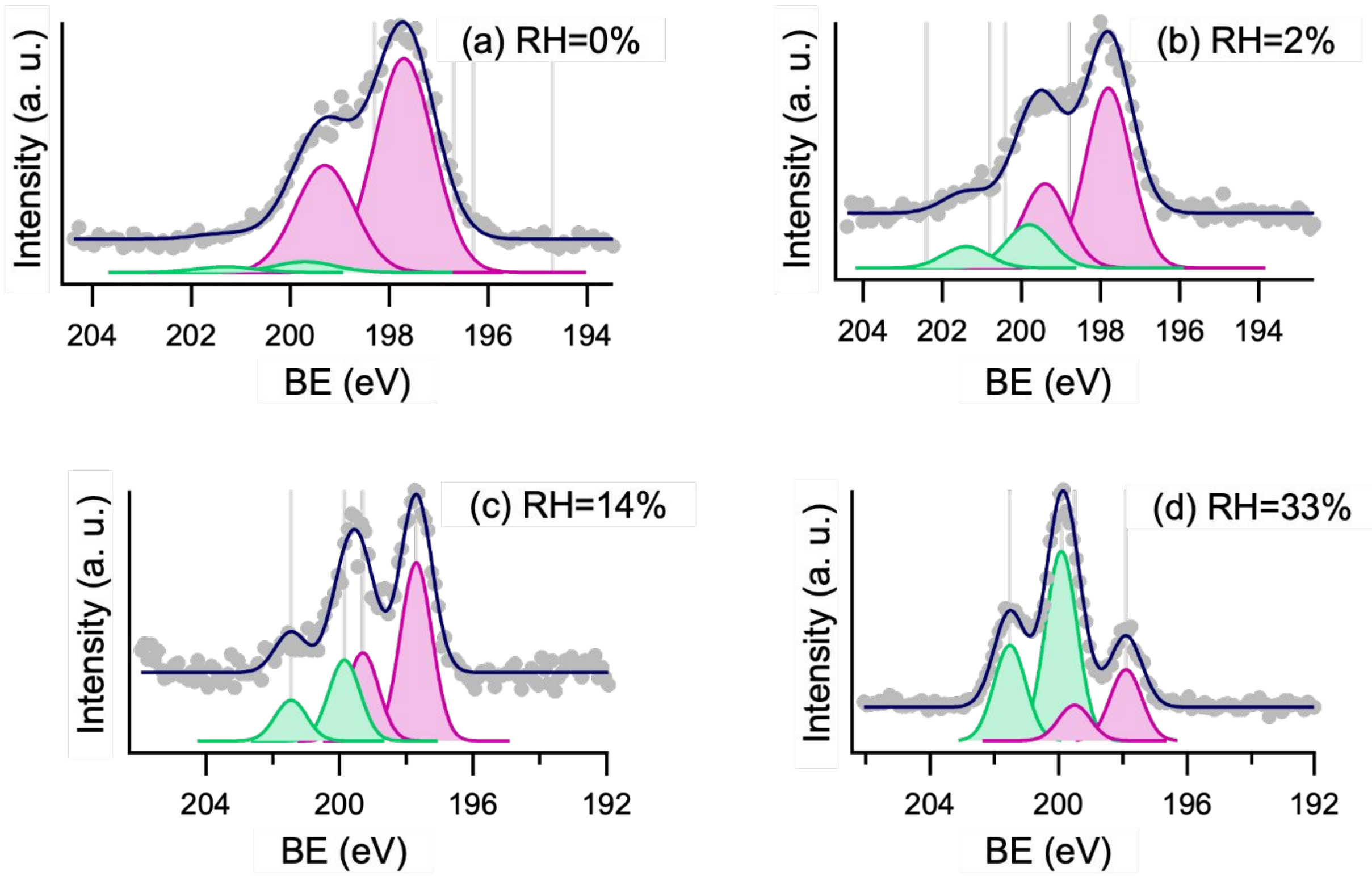


**Figure S1.** High-resolution Cl 2p XPS spectra of $MgCl_2$ measured under varying RH conditions: (a) 0%, (b) 2%, (c) 14%, and (d) 33%. Experimental data (gray circles) are shown together with the fitted spectra (black lines) and individual peak components corresponding to $Cl^-$ (magenta) and HCl (green). The evolution of spectral features with increasing RH indicates the emergence of an additional chlorine-containing species associated with hydration.

37

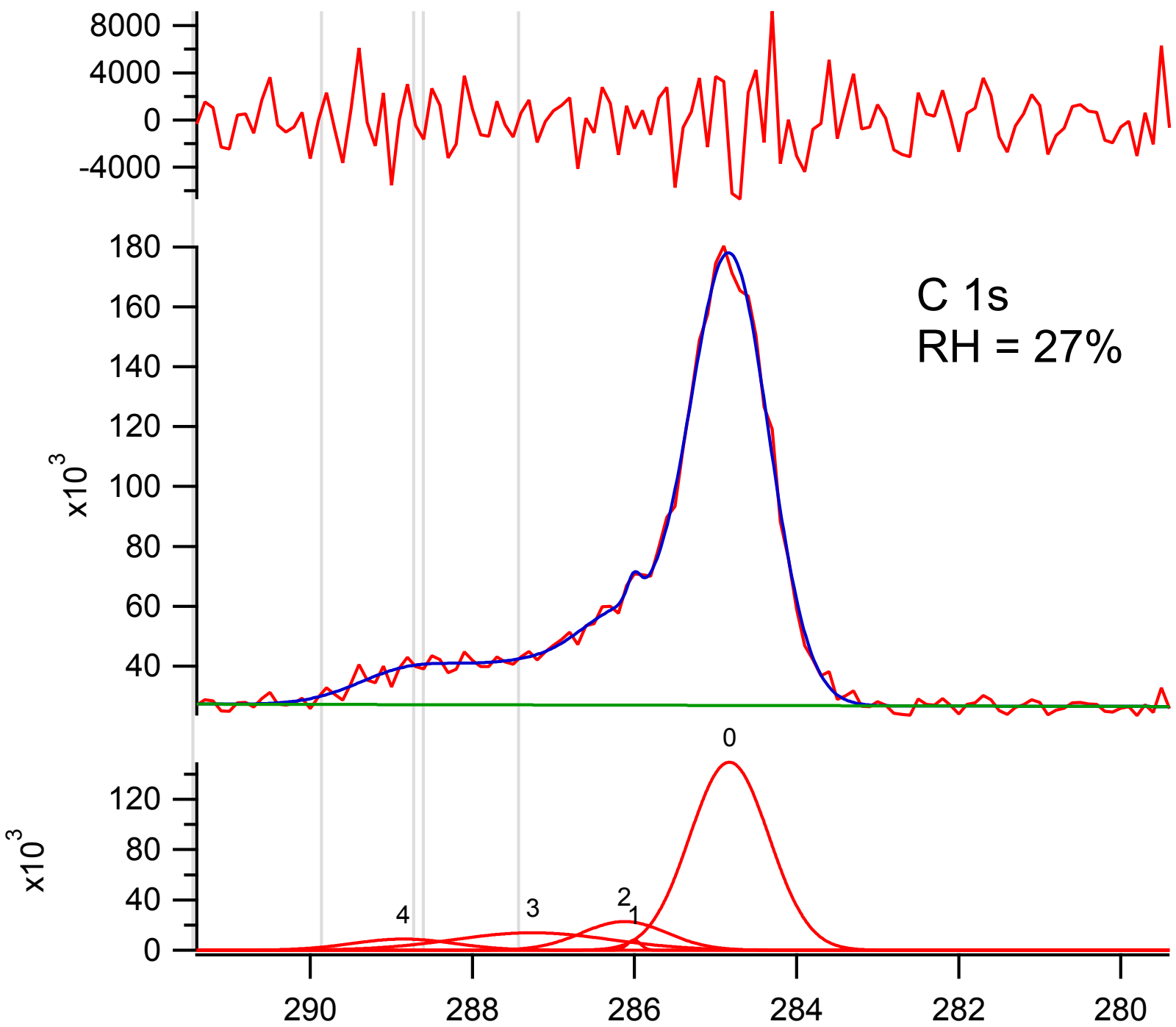


**Figure S2. High-resolution C 1s APXPS spectrum of the natural salt surface measured at RH = 27% together with the peak deconvolution.** The experimental spectrum (red), overall fit (blue), and individual fitted components are shown. The spectrum is dominated by the C-C/C-H component, with minor contributions from oxygenated carbon species (C-O, C=O, and O-C=O) characteristic of adventitious carbon. No distinct carbonate component is observed in the carbonate binding-energy region (~290 eV), indicating the absence of significant surface enrichment of carbonate species under the present experimental conditions. The residuals are shown at the top of the figure. The C-C/C-H component at 284.8 eV was used for binding-energy calibration.

**Table S1.** Cl 2p fitting parameters for the representative hydrated spectrum at RH 36% shown in Figure 2c.

| Species | Cl 2p component | Binding energy (eV) | FWHM (eV) | Area | Line shape |
|---|---|---|---|---|---|
| $Cl^-$ | 2p3/2 | 198.1 | 1.41 | $1.0938 \times 10^6$ | Gaussian |
| $Cl^-$ | 2p1/2 | 199.5 | 1.41 | $5.4691 \times 10^5$ | Gaussian |
| HCl | 2p3/2 | 200.1 | 1.38 | $1.0681 \times 10^6$ | Gaussian |
| HCl | 2p1/2 | 201.7 | 1.52 | $5.4409 \times 10^5$ | Gaussian |

Linear background was used. The $Cl^-$ doublet has a spin-orbit splitting of 1.4 eV and an area ratio of 2:1. The HCl doublet has a spin-orbit splitting of 1.5 eV and an area ratio of 2:1. Fit quality metrics for the representative spectrum at RH 36%: $\chi^2 = 7.58 \times 10^9$; total fitted points = 121. Residuals exhibited no systematic trends and were randomly distributed around zero.

## Experimental time series and transient-region definition

Figures S3-S5 show the time evolution of RH, temperature, chamber pressure, and Cl 2p spectra during RH cycling experiments. The shaded regions indicate transient periods immediately following changes in RH or temperature, during which the Cl 2p spectral shape and intensity continued to evolve. These spectra were therefore not used as representative steady-state spectra in the main-text discussion.

Because the salt film is a multicomponent and partially deliquescing system, apparent stabilization of the gas-phase RH does not necessarily imply instantaneous equilibration of the condensed phase. Water uptake can be influenced by water-vapor transport to the sample, diffusion through hydrated or partially deliquesced surface layers, dissolution/recrystallization kinetics, and lateral redistribution within the salt film. The shaded regions therefore provide an operational definition of non-equilibrated periods based on the observed spectral evolution rather than on RH stabilization alone.

Representative spectra used in the main text were selected only after both the imposed RH and the Cl 2p spectral response had stabilized within experimental uncertainty.

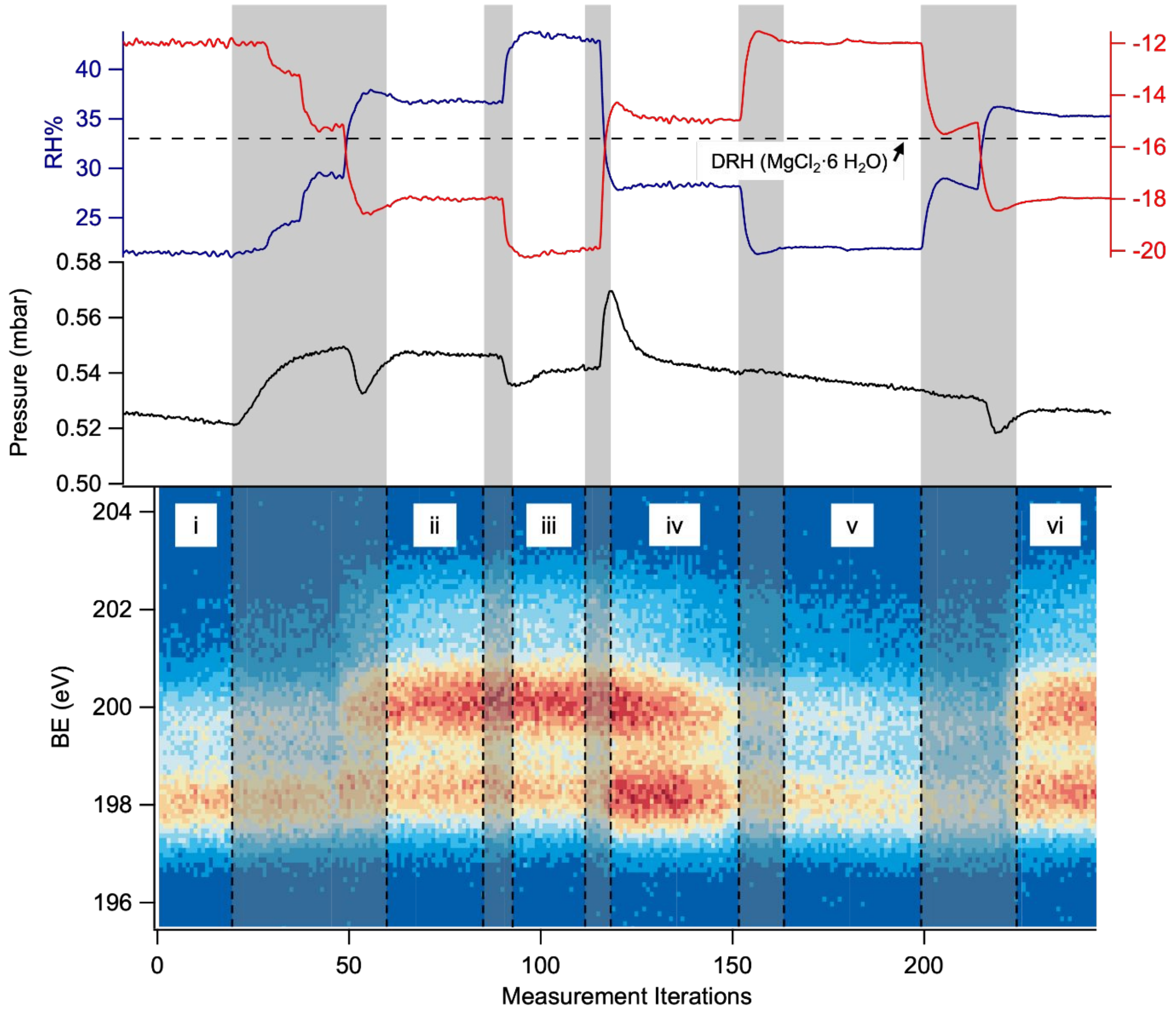


**Figure S3.** Time series of experimental conditions and corresponding Cl 2p XPS spectra during RH cycling. The upper panel shows the evolution of RH (blue), temperature (red), and chamber pressure (black) as a function of measurement iteration. The dashed line indicates the DRH of $MgCl_2{\cdot}6H_2O$ (~33% RH), used as a reference for $MgCl_2$-controlled hydration. Shaded regions denote transient periods following changes in RH or temperature, during which the Cl 2p spectra continued to evolve and were therefore excluded from steady-state interpretation. The lower panel displays the time-resolved Cl 2p spectra, where the color scale represents spectral intensity. Roman numerals indicate distinct RH stages discussed in the main text.

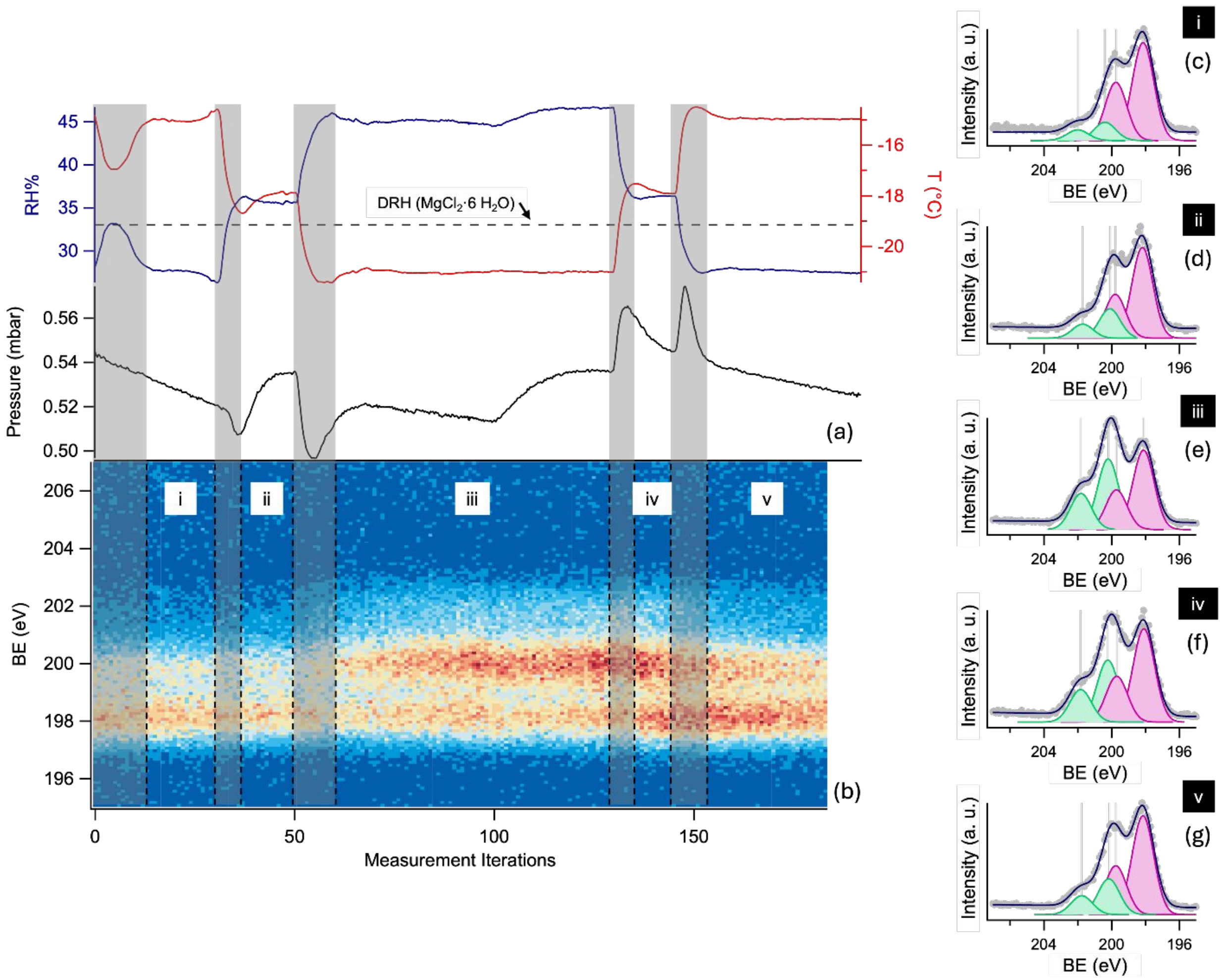


**Figure S4.** Time-resolved APXPS measurements during increasing RH. The upper panel shows the evolution of RH (blue), temperature (red), and chamber pressure (black) as a function of measurement iteration. Representative Cl 2p spectra with peak fitting ($Cl^-$ in magenta and HCl in green) are shown for selected stages (i-v). Shaded regions, if shown, indicate periods of spectral evolution following changes in RH or temperature and were not used for steady-state interpretation.

74

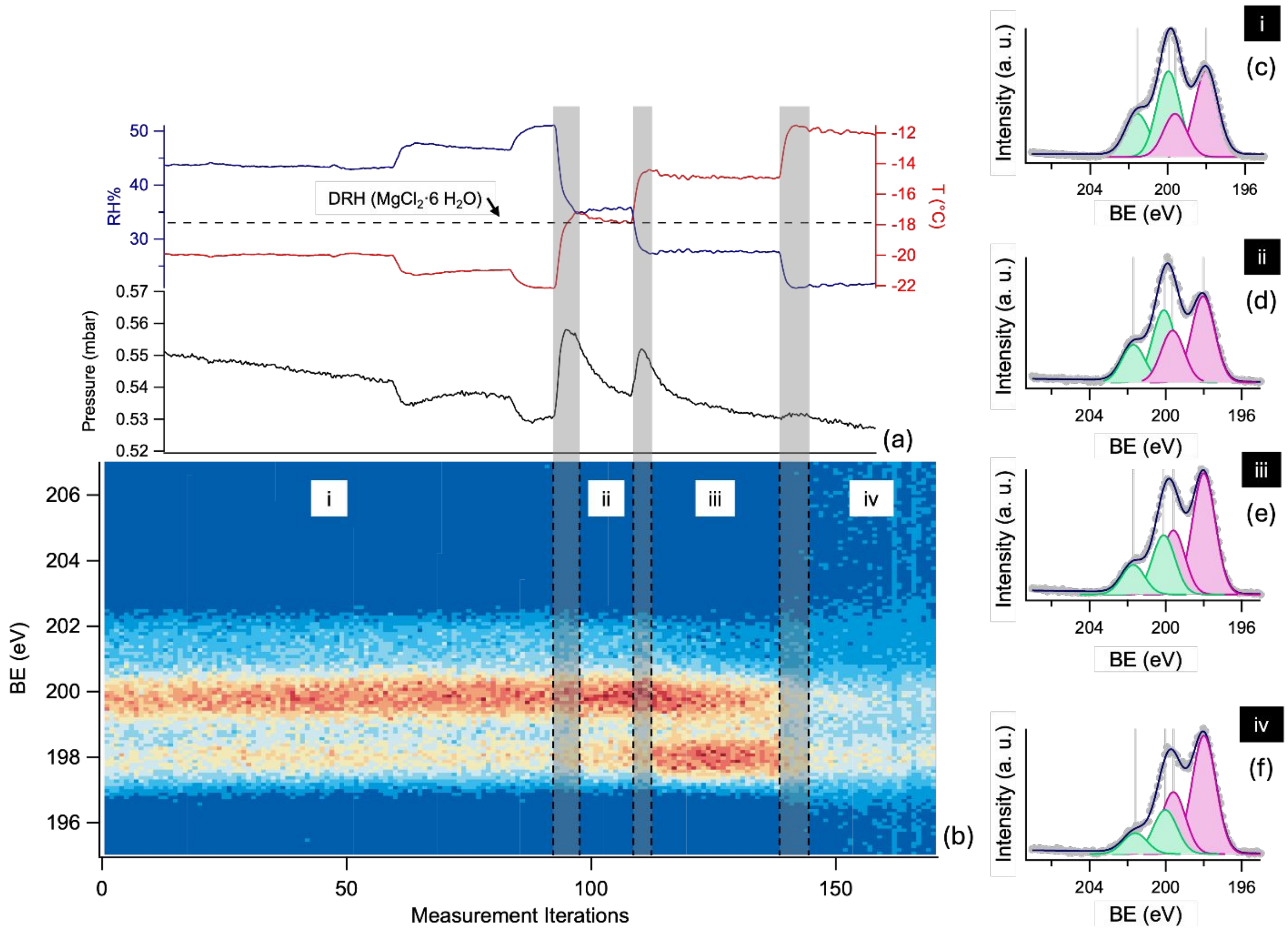


**Figure S5.** Time-resolved APXPS measurements during decreasing RH for the same sample in Figure S3. Shaded regions, if shown, indicate periods of spectral evolution following changes in RH or temperature and were not used for steady-state interpretation.
